# Potential and Design Parameters of Polyvinylidene Fluoride in Gear Applications

**DOI:** 10.3390/polym15214275

**Published:** 2023-10-31

**Authors:** Enis Muratović, Adil Muminović, Muamer Delić, Nedim Pervan, Adis J. Muminović, Isad Šarić

**Affiliations:** Department of Mechanical Design, Faculty of Mechanical Engineering, University of Sarajevo, 71000 Sarajevo, Bosnia and Herzegovina; muratovic@mef.unsa.ba (E.M.); muminovic@mef.unsa.ba (A.M.); delic@mef.unsa.ba (M.D.); adis.muminovic@mef.unsa.ba (A.J.M.); saric@mef.unsa.ba (I.Š.)

**Keywords:** PVDF polymer, gear application, failure modes, lifespan, coefficient of friction, wear coefficient, tribological compatibility

## Abstract

(1) Background: With the ever-increasing number of polymer materials and limited data on polymer gear calculations, designers are often required to perform extensive experimental testing in order to establish reliable operational data for specific gear applications. This research investigates the potential of a Polyvinyldene fluoride (PVDF) polymer material in gear applications, considering various loading conditions and different types of gear transmission configurations, including both self-mated mesh and steel/PVDF mesh. (2) Methods: PVDF gear samples were tested on a specially designed test rig that enables active torque control and temperature monitoring in order to obtain the necessary design parameters and failure modes. Each test for certain load conditions was repeated five times, and to fully investigate the potential of PVDF gear samples, comparative testing was performed for Polyoxymethylene (POM) gear. (3) Results: Tribological compatibility, tooth load capacity, and lifespan assessment, along with the types of failure, which, for some configurations, include several types of failures, such as wear and melting, were determined. Temperature monitoring data were used to estimate the coefficient of friction at the tooth contact of analyzed gear pairs, while optical methods were used to determine a wear coefficient. (4) Conclusions: The tribological compatibility of polymer gear pairs needs to be established in order to design a gear pair for a specific application. PVDF gear samples mated with steel gear showed similar lifespan properties compared to POM samples. Temperature monitoring and optical methods serve as a basis for the determination of the design parameters. PVDF is an appropriate material to use in gear applications, considering its comparable properties with POM. The particular significance of this research is reflected in the establishment of the design parameters of PVDF gear, as well as in the analysis of the potential of the PVDF material in gear applications, which gives exceptional significance to the current knowledge on polymer gears, considering that the PVDF material has not previously been analyzed in gear applications.

## 1. Introduction

Polymer gears play an increasingly important role in many new applications. In the past, these gears were typically used in low-demand applications, but with the development of new high-performance thermoplastic materials, they are being increasingly used in liability critical systems, such as control and braking systems [1]. With the ever-demanding applications of polymer gears, and taking into consideration the rate of development of new polymer materials and the number of commercially available materials, it is not an easy task to design a polymer gear for a specific application [2], especially taking into account the various number of parameters that need to be determined via time-consuming and expensive tests [3,4,5].

Although many types of research cover polymer gear problematics [3,4,5], there are many polymer materials, such as PVDF, that need to be characterized in order to establish reliable operational data for the polymer gear design process [1,6,7,8]. PVDF material is classified as an engineering thermoplastic fluoropolymer with excellent thermal, mechanical, and chemical characteristics. The performance of PVDF in numerous applications has been studied, with a focus on the tribological and mechanical properties [9,10,11,12,13]. The main objective of this research is to study the potential of PVDF in gear applications since no research on this topic has been conducted yet.

The polymer gear design process mostly relies on the VDI 2736 guidelines, which represent one of the few guidelines with available data for specific gear operations [14,15,16,17]. Even though these guidelines are extensively used, they have a lot of shortcomings, such as limited gear endurance data, which are generally introduced for polyamide (PA) and POM gears, resulting in inadequate support when it comes to determining the design parameters of other polymer materials [8,18]. The use of VDI 2736 guidelines is even more limited in situations where the design process is conditioned with wear coefficients since the coefficients in the guidelines are obtained from classic tribological tests, i.e., pin-on-disc experiments [18]. These coefficients, which are also introduced for a limited number of materials, cannot be used for gear design in real applications due to inevitable differences in the results obtained from tribological and real-scale gear testing [19].

Tribological and mechanical tests offer information about material properties [20,21], but in order to obtain parameters relevant to gear design in a specific application, gear testing is required [2]. For this purpose, many researchers have developed specially designed test rigs for polymer gears that enable the experimental examination of specific values that help researchers to precisely describe and determine many parameters related to gear design and the type of failure, such as the coefficient of friction at the meshing zone, tooth root load capacity, tooth flank load capacity, wear coefficient, bulk temperature, flash temperature, lifespan at the certain load level, noise development, etc. [20,21,22,23,24,25,26]. The difficulty in identifying these parameters is reflected in the uniqueness of a gear design for a specific application since gear testing is conducted exclusively for certain geometries, materials, and load conditions [27]. Furthermore, even more difficulties occur within the limitations of test rigs, and the mechanical and tribological properties of polymer materials are greatly affected by temperature changes [3,8,20,21]. Test rigs are usually designed with the possibility of performing full-time tests as well as incremental load tests, i.e., step tests [3,8,26]. Full-time tests provide lifespan data of a polymer gear under certain load conditions that correspond to gear application conditions [7]. Time-consuming full-time tests are often substituted with incremental load tests, which provide polymer gear endurance limits in a much shorter time with a reduced number of gear samples and tests required [8,28]. Incremental load tests are carried out on a certain load level for a predetermined period, which is usually defined as the time necessary for a polymer gear to reach thermal stability [3,8].

One more important aspect of the polymer gear design process is the failure mechanism. The main types of failure mechanisms include temperature overload, i.e., melting, wear, and fatigue [3,6,8,27]. The type of failure that is most likely to occur depends on many factors, which are frequently attributed to material pairing, load conditions, and lubrication regimes [2]. Material pairing is often referred to as tribological compatibility between materials of pinion and gear in a mesh. With the enormous number of polymer materials, it is necessary to perform preliminary tribological tests to ensure that material pairing with substandard performance is avoided [29,30]. Tribologically incompatible material pairing often leads to premature melting failure in the early stages of testing and high wear rates even at low load levels [8,24]. The impact of the load level and the lubrication regime are tightly connected to the polymer gear performances and the reasoning behind these operational outcomes is even further mystified by the polymer gear geometry and material properties [31,32]. As many researchers have established, an identical gear pairing at higher loads fails due to temperature overload in the case of dry-running, while in the case of lubrication, failure occurs due to fatigue or lubrication-induced pitting [1,27]. In the case of dry running, the temperature overload is attributed to friction generated at the surfaces of meshing flanks, contrary to the lubrication conditions where the flank surfaces are separated by the lubricant film, which results in a lower temperature load due to reduced friction at the tooth contact and heat removal by the lubricant itself [33]. The influence of the load on the thermal behavior of the polymer gears is also specified by the rotational speed of the gear pair [34]. At the same load conditions, some configurations experience greater thermal loads in the lower rotational speed conditions where the prolonged contact time of the tooth flanks, i.e., sliding of the flank surfaces leads to an increase in frictional losses [35,36].

The wear failure is one of several failure mechanisms experienced at the tooth flank meshing interface, which operates under the combined motion of sliding and rolling. Besides the direct loss of material that leads to tooth flank degradation, surface wear induces a change in the vibration and noise properties of the gear pair, since the gear meshing is very dependent on the tooth surface geometry [25]. Considering the typical engagement of a polymer gear with a metal pinion, wear-induced failure is to be expected due to the hardness difference between contacting surfaces where the steel pinion abrasively wears the softer polymer surface. This leads to a reduced tooth cross-section with compromised bending stiffness, which eventually results in tooth fracture [2,29]. Polymer gear engagement and wear mechanisms can be divided into several interdependent phases according to the intensity of the wear rate during the operation. The beginning of the gear pair engagement, i.e., the running-in phase, is distinguished by high wear rates over a short period caused by the penetration of the metal surface over the micro-asperities of the polymer gear flank. The polymer material, which is removed by the shear mechanism, forms a transfer layer leading to the establishment of the linear wear phase caused by the third-body abrasion [37], with a low progressive wear rate over many working cycles [3,6]. The final phase occurs at the end of the polymer gear service life, with the thermal overload significantly increasing the wear rate. During these phases, as the flank contact topography constantly changes, the tooth flank surface experiences severe changes in contact conditions regarding contact pressure distribution and relative sliding [38,39].

As emphasized, polymer gears happen to fail due to different types of defects. Although experimental testing provides information about the type of failure that should be expected in a real application, the gear design process often needs to take into account the fact that several types of failure mechanisms may occur. To positively influence one or more properties or to specifically modify them, the base polymer material is often reinforced with glass or carbon fibers [34]. These fiber reinforcements are usually added to improve mechanical properties such as Young’s modulus. Also, a similar approach can be used to positively influence the tribological properties of the tooth flank. The thermal characteristics and wear of the tooth flank may be optimized by reducing the frictional properties with additives such as graphite or PTFE (polytetrafluoroethylene). This way, the coefficient of friction can be adjusted, i.e., reduced, along with the flash temperature at the tooth contact, utilizing friction-reducing fillers [1]. Therefore, expected failure mechanisms and gear application often require the previously mentioned adjustments, where, in order to achieve an optimal design, it is necessary to find an optimal percentage of fibers or fillers, which on the one hand depends on the type of improvement which is required for gear design, and on the other must not compromise mechanical properties [40,41].

With the wide scope of different polymer materials and limited data available, the polymer gear design process can only be performed through experimental testing of gear drives, which leads to the reliable use of polymer gears in real-life applications [25,31,42]. Considering the PVDF polymer material [43,44] whose potential in gear applications was tested, there is currently no available data for the design process of the PVDF gears; furthermore, no research on this topic has been detected by the authors. For this reason, taking into consideration the good mechanical properties of the PVDF polymer as well as the friction and wear characteristics, PVDF gears have been tested. Performed testing included self-mated and steel/PVDF mesh under various loading conditions for the case of dry running. The objective of this research is to obtain data related to the tribological compatibility of the material pairing, the coefficient of friction at the tooth contact, tooth load capacity data, and failure mechanisms. The special focus of the research is directed toward the determination of the wear coefficients for the steel/PVDF gear pairs, as it is quite certain that abrasive wear occurs on the polymer gear tooth flank.

## 2. Materials and Methods

### 2.1. Data on Tested Gears

The vast majority of polymer gears are manufactured by injection molding, especially in cases of mass production where the injection molding process is the most cost effective regarding individual gear costs. Due to the smaller number of gear samples and desired gear quality, the gear samples presented in this research are manufactured by hobbing. Testing included a self-mated mesh of polymer gears and steel/polymer mesh. Steel gears were made from C45 steel, while polymer gears were made from PVDF (Arkema, Colombes, France) material with properties according to Table 1.

To fully explore the potential of PVDF in gear applications, parallel testing was conducted on the gear samples made out of POM (DuPont, Neu-Isenburg, Germany), which is one of the most commonly used polymer materials for gears. POM material properties are shown in Table 2.

Table 3 shows the main chemical properties of the materials used for the gears.

To fully investigate the mechanical properties of used polymer materials, which degrade as the operating temperature increases, Table 4 shows the comparative data for PVDF and POM materials at the temperature of 120 °C and corresponding heat deflection temperatures of 150 °C and 140 °C, respectively [54,55].

As shown in Table 4, the mechanical properties of the polymer materials degrade with temperature. This has a significant effect on the load capacity of polymer gears regarding tooth root stress and tooth flank pressure. With the decrease in tensile modulus due to elevated temperature, polymer gear teeth are prone to deforming at lower load levels, i.e., the permissible tooth root stress is lower. At the same time, the temperature-dependent tensile modulus reduces the tooth flank load-carrying capacity as the prolonged contact area reduces the normal load. Although the normal load is reduced, the meshing of polymer gears is disrupted as adhesive contact conditions cause surfaces to stick, which contributes to more intense wear of the teeth, i.e., temperature-induced wear.

The geometry of the tested gear samples was the same for both PVDF and POM gears. The C45 steel pinion also had the same geometric parameters except for the face width, which was 1 mm wider. The reason for this is the obvious difference in the mechanical and thermal properties of the polymer and metal gears. With the mechanical properties of the polymer gears being affected by the temperature rise and considering the gear size and thermal conductivity of the polymer materials, the polymer gear geometry shows a tendency to expand, especially at the face width direction where the tooth flank experiences the effects of the flash temperatures during the engagement. Also, Young’s modulus of polymer gear, which is many times lower compared to that of steel, has a great impact on the tooth deflections that cause contact flattening, which contributes to face width expansion. This way, considering the expansion of polymer gears in mesh with steel pinion, a greater uniform transmission of the rotary motion is achieved along with better heat dissipation due to the extended face width of the steel pinion. The basic geometric parameters of the tested polymer gears are shown in Table 5.

The specially developed test rig was designed for larger gear samples, where the geometry of tested polymer gear samples was defined according to the test rig’s limitations.

### 2.2. Polymer Gear Test Rig

**Figure 1:**
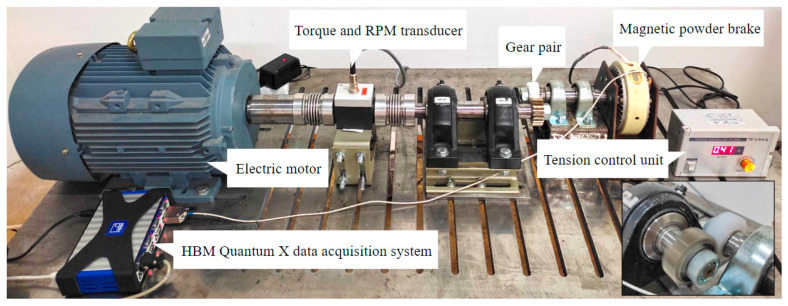
Polymer gear test rig.

Testing is performed on an open-loop test rig shown in Figure 1. The power is supplied through an electric motor (Marathon Electric HJA-IE2 132 M, Regal Rexnord Corporation, Wausau, WI, USA) which is controlled by a frequency regulator (EN600-4 T 0075G/110P, Shenzhen Encom Electric Technologies CO, Shenzen, China). The torque transducer (HBM T20WN T153040, Hottinger Baldwin Messtechnik GmbH, Darmstadt, Germany), which is used for the measurement of torque and angular velocity, is connected to the HBM Quantum X data acquisition system.

**Figure 2:**
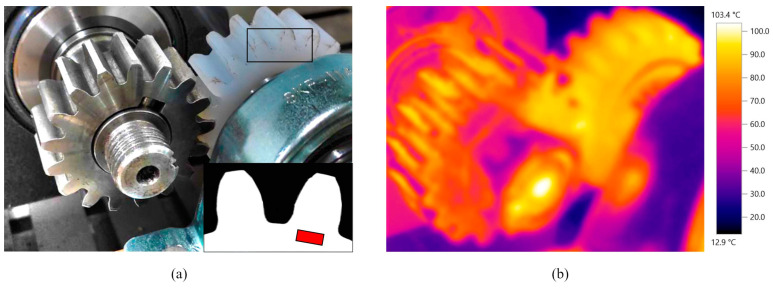
Temperature monitoring: (**a**) reference area at the tooth root; (**b**) thermal view.

The electric motor is connected, via couplings and bearing units, to the shaft on which the pinion is mounted. The gear is mounted on the other shaft, which is connected, via bearing units, to the magnetic powder brake (FZ-12-K TB-200S, Yun Duan, Taiwan, China) with a maximum achievable torque of 12 Nm. The amount of necessary torque is precisely adjusted with the tension controller unit (potentiometer). The developed test rig allows adjustment of the position of the second shaft in a horizontal direction, which enables the testing of gear samples of different sizes. The initial torque of the bearing units and two parallel shafts is neglected, i.e., zeroed with the catmanEasy 5.3.1 software of the Quantum X data acquisition system. The temperature of the tested gears is monitored with the thermal camera (Testo 865, Testo SE, Titisee-Neustadt, Germany). The average gear bulk temperature is determined by monitoring a temperature increase over a small area located at the tooth root, as shown in Figure 2a. The region of this area is adjusted in the IRSoft 4.8 thermal camera software. The emissivity of the polymer gear materials is established before the tests are conducted and set to a value of 0.95, which remains constant during the tests. The monitored gear sample temperature is obtained with the thermal view shown in Figure 2b.

### 2.3. Testing Conditions

The objective of the tests was to explore the potential of the PVDF material in gear applications. To achieve that, parallel testing was performed on POM gear samples of the same geometry at the same load conditions. The tests conducted included the following transmission configurations:

1. Incremental load tests (step tests) of PVDF self-mated gear samples;
2. Incremental load tests (step tests) of POM self-mated gear samples;
3. Lifetime tests of steel pinion and PVDF gear samples;
4. Lifetime tests of steel pinion and POM gear samples.

The purpose of the incremental load tests for the configurations of the self-mated contact is to quickly establish the tribological compatibility of the polymer materials at the different load levels. This type of testing was used because it is easier to obtain the necessary information about the approval or rejection of gear materials than performing the test at the single load level. The starting load of the incremental tests, considering the gear geometry, was set to 1 Nm. The load for each step was increased by 0.5 Nm until the failure occurred on one of the gears. The duration of the conducted tests at one load level, i.e., step, was specified at 2 × 10^5^ cycles, which is a recommended period that ensures gear temperature stability at a certain load level. As is customary in gear transmission, a metal pinion is often paired with the polymer gear due to the heating dissipation effect, which is much better compared with a pair of meshing polymer gears. Due to this effect, a much greater lifespan of polymer gear is expected when paired with a metal pinion, since the chances of thermal failure are almost completely avoided and thermal effects on the mechanical and tribological properties of polymer material differ significantly, i.e., they will not degrade as much compared to a pair of meshing polymer gears. Taking this into account, as well as the gear sizes and maximum torque of 12 Nm that can be provided by the magnetic powder brake, lifetime testing at constant load and constant speed was performed for the steel/polymer pairing configurations. In this case, the gear samples were tested, considering the geometry and real-life applications [35], at three different load levels, i.e., 4 Nm, 5 Nm, and 6 Nm. Before these tests, a series of tests were performed on a 2 Nm load level where the polymer gears achieved long lifespans. As is the case in numerous applications for polymer gears to perform in non-lubricated conditions, which represents one of their main advantages, the gear samples were also tested in the most challenging operating conditions, i.e., dry friction conditions with no lubrication between the pinion that supplies power to the open-loop system and the gear connected to the magnetic powder brake. Incremental load tests and lifetime tests were performed with the gear samples operating at a rotational speed of 1000 rpm, which was specified according to the operational parameters of the magnetic powder brake. Each test was repeated five times to obtain reliable data for gear design calculations. The basic testing parameters of polymer gears at the pitch diameter are shown in Table 6, along with the minimum and maximum values of specific sliding. The minimum and maximum values of specific sliding, due to pinion and gear geometry, were the same for both gear and pinion, with the latter being located on different points of engagement, i.e., the root area and the tip area.

### 2.4. Gear Calculations

To provide data for the load-carrying capacity of polymer gears, considering obvious differences in the material properties of metallic and polymer gears, VDI 2736 guidelines were used. The purpose of these guidelines, especially in cases of designing a new gear pair, is reflected in the necessary data on the coefficient of friction, root fatigue, rolling contact fatigue, etc. The VDI 2736 calculation models, with the previously mentioned data obtained on several iterations of load levels, enable the use of the test data on standard gear pairs in applications with similar geometry and load conditions. This way, the process of designing a new drive is based on reliable operational data, which reduces the time and cost of developing a new drive. In other words, the VDI 2736 guidelines enable the calculation of safety and critical values regarding tooth base break, tooth flank damage, and tooth distortion [15]. With the specific values obtained, it is possible to determine which parameters lead to gear failure, i.e., which failure mechanism is most likely to occur, in order to take preventive measures in the design process.

Tooth root stress calculation was performed for different torque levels according to Equation (1), which is implemented in the VDI 2736 guidelines [15]. Torque levels for which the failure occurs were not included.

(1) $$\sigma_{F}=K_{F}\cdot Y_{Fa}\cdot Y_{Sa}\cdot Y_{\varepsilon }\cdot Y_{\beta }\cdot \frac{F_{t}}{b\cdot m_{n}}.$$

According to the gear sample geometry [15], the coefficients and sizes were:

- $K_{F}=1$—Tooth root factor (chosen coefficient is based on DIN 3990 [57]);
- $Y_{Fa}=3.075$—Form factor;
- $Y_{Sa}=1.757$—Stress correction factor;
- $Y_{\varepsilon }=0.757$—Contact ratio factor;
- $Y_{\beta }=1$—Helix angle factor;
- $F_{t}$—Nominal tangential force (based on load level i.e., torque);
- b = 20 mm—Face width;
- $m_{n}$ = 3 mm—Gear sample module.

To fully define the tooth load capacity, it is necessary to define the flank pressure according to Equation (2), which follows the VDI 2736 model [15]:

(2) $$\sigma_{H}=Z_{E}\cdot Z_{H}\cdot Z_{\varepsilon }\cdot Z_{\beta }\cdot \sqrt{\frac{F_{t}\cdot K_{H}}{b_{w}\cdot d_{1}}\cdot \frac{u+1}{u}}.$$

Flank pressure calculations also do not consider the critical torque values. For the gear geometry and material properties, the coefficients and sizes were:

- $Z_{EPVDF}=19.05$—Elasticity factor for self-mated PVDF gear samples;
- $Z_{EPOM}=22.54$—Elasticity factor for self-mated POM gear samples;
- $Z_{H}=2.495$—Zone factor;
- $Z_{\varepsilon }=0.814$—Contact ratio factor;
- $Z_{\beta }=1$—Sprial angle factor;
- $b_{w}=20\;$ mm—Common face width;
- $K_{H}=1$—Factor for tooth flank loading;
- $d_{1}=51$ mm—Pinion diameter;
- u=1—Gear ratio.

The elasticity factor $Z_{E}$ is extremely important for calculating the bearing capacity of the tooth flank and must be defined for each material, i.e., mesh. According to VDI 2736 guidelines, the elasticity factor $Z_{E}$ is represented by Equation (3):

(3) $$Z_{E}=\sqrt{\frac{1}{\pi \cdot \left(\frac{1-\upsilon_{1}^{2}}{E_{1}}+\frac{1-\upsilon_{2}^{2}}{E_{2}}\right)}},$$

with a tensile modulus according to Table 1 and Table 2 and Poisson’s ratio for PVDF and POM material of $\upsilon_{PVDF}=\upsilon_{POM}=0.35$.

Since polymer material properties such as modulus of elasticity and strength are highly temperature dependent, the temperature distribution during the gear pair engagement has to be known to avoid undesired failures such as melting or fatigue damage induced by elevated temperatures [1]. The root temperature of the polymer gear, which was monitored during the tests, is often considered the bulk temperature, which can be expressed by Equation (4):

(4) $$\vartheta_{Fu\beta }=\vartheta_{0}+P\cdot \mu \cdot H_{v}\cdot \left(\frac{k_{\vartheta ,Fu\beta }}{b\cdot z\cdot {\left(v_{t}\cdot m_{n}\right)}^{0.75}}+\frac{R_{\lambda ,G}}{A_{G}}\right)\cdot {ED}^{0.64}.$$

This equation is generally used to evaluate the coefficient of friction between the meshing flanks for specific material combinations [2,8,15,18,29]. The coefficient of friction was evaluated using the following values:

- $\vartheta_{0}=20$ °C—Ambient temperature;
- P—Transmitted power (based on load level, i.e., torque);
- $H_{v}=0.632$—Degree of tooth loss;
- $k_{\vartheta ,Fu\beta }=2100\frac{K\cdot {\left(\frac{m}{s}\right)}^{0.75}\cdot {mm}^{1.75}}{W}$—Heat transfer coefficient;
- b=20 mm—Face width;
- z=17—Number of teeth;
- $v_{t}=2.67$ m/s—Tangential velocity (for 1000 rpm);
- $m_{n}=3$ mm—Gear sample module;
- ED=1 (100%)—Relative tooth engagement time.

The values $R_{\lambda ,G}$ and $A_{G}$ were neglected from Equation (4) since they represent heat transfer resistance and the heat-dissipating surface of the housing, which are not applied for non-enclosed mechanisms.

The values of specific factors and coefficients presented in Equations (2) and (4) were specified for the polymer self-mated configurations. Since this research also covers steel/polymer engagement, the appropriate values concerning this engagement are emphasized throughout the manuscript.

## 3. Results

### 3.1. Incremental Load Test Results

#### 3.1.1. Failure Mechanisms

**Figure 3:**
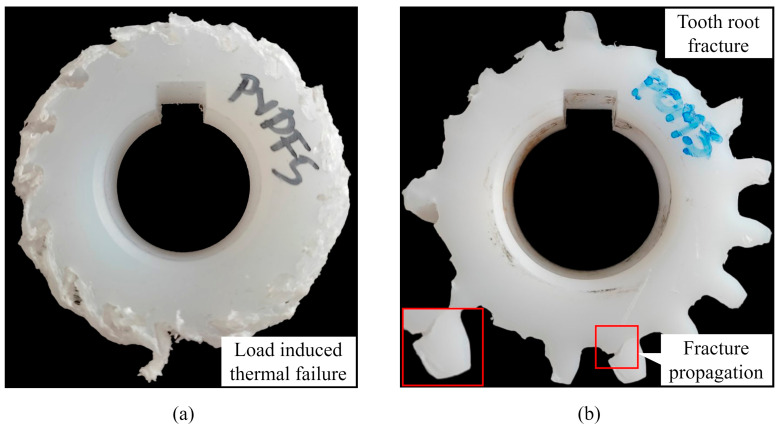
Failure mechanisms: (**a**) PVDF self-mated gears; (**b**) POM self-mated gears.

Incremental load tests revealed different failure mechanisms of PVDF and POM gears in self-mated contact. PVDF gears, with the previously described step test setup, reached a maximum torque of 9 Nm. At each step, load-level PVDF gears experienced the simultaneous effects of melting and fatigue. The melting effect was more distinctive at higher load levels (above 4 Nm). Temperature stability criteria were fulfilled at each load level below 9 Nm. At the 9 Nm load level, due to a dramatic increase in temperature, gears experienced a complete thermal overload, as shown in Figure 3a. Therefore, the value of 9 Nm was defined as a critical value, i.e., the maximum endurance limit of the PVDF gears in self-mated contact. Gear tooth flanks showed no signs of wear during the tests.

**Figure 4:**
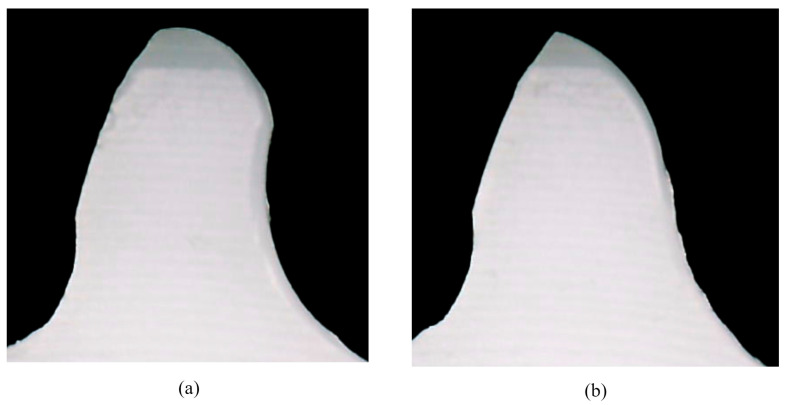
The excessive wear rate of the self-mated POM gears: (**a**) 2 Nm load level; (**b**) 4 Nm load levels.

POM gears reached a maximum torque of 8 Nm. At each load level, POM gears experienced a wear failure mechanism. The wear rate was high even at the lower load levels (2 Nm). The wear rate was established with a visual inspection, i.e., a digital microscope (NB-MIKR-500). Due to the high wear rate, the tooth flank experienced severe changes in profile geometry, resulting in a reduced cross-section with compromised bending stiffness, which eventually led to the tooth root fracture, as shown in Figure 3b. The initiation of the tooth root fracture was located at the dedendum area of the tooth flank and appeared in the form of a crack that propagated over the tooth root diameter area, causing the fracture. At higher load levels (above 4 Nm), even though the temperature stability criterion is fulfilled, reduced cross-section leads to reduced heat dissipation from the contact area. This causes thermally induced plastic deformations of the gear teeth, which can be noticed in Figure 3b. Figure 4a shows the tooth profile of the POM gears under a load level of 2 Nm. In the early stages of load incremental testing, severe wear was observed at the tooth root. With the increased load level, as the contact conditions changed, wear propagation was more significant at the pitch point region and the addendum area, as shown in Figure 4b for a load level of 4 Nm.

#### 3.1.2. Design Parameters

Table 7 presents the evaluated design parameters regarding tooth load capacity and estimated coefficients of friction for the analyzed PVDF and POM gear samples in the self-mated mesh.

### 3.2. Lifetime Tests Results

#### 3.2.1. Failure Mechanisms

**Figure 5:**
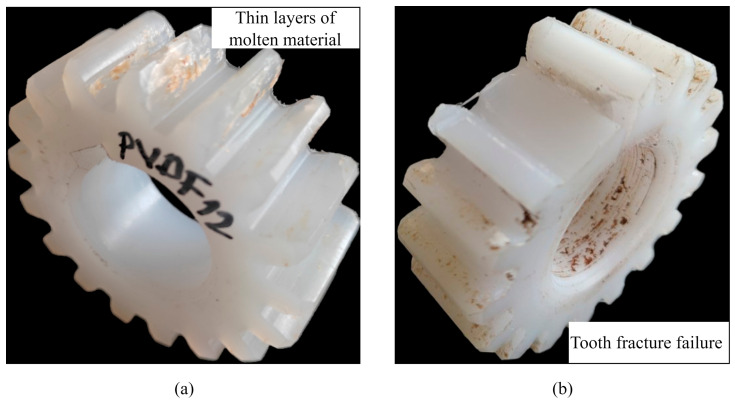
Wear failure mechanisms: (**a**) a layer of molten material acts as a lubricant in steel/PVDF mesh; (**b**) POM gear tooth fracture due to reduced tooth thickness.

PVDF and POM gears mated with steel pinion failed due to wear. Such a failure mechanism is typical for steel/polymer mesh due to differences in the hardness of contacting surfaces where abrasive wear, especially in the case of dry running, is expected. For both materials, the wear rate reached the point where the tooth cross-section was reduced to a critical size, which resulted in a tooth fracture. However, the wear mechanism of the PVDF gears had one difference. During the engagement of the PVDF gears with the steel pinion, a thin brown film covered the surfaces of the tooth flanks, as shown in Figure 5a. This was caused by frictional losses at the gear contact, which led to the melting damage of the PVDF material. This phenomenon, also observed by other researchers [3], actually has a positive effect on the tooth flank because the thin layers of molten material function as an internal lubricant, which decreases the wear rate. The typical wear-induced tooth fracture, with negligible thermal effects of the POM gear, is shown in Figure 5b.

#### 3.2.2. Lifespan

**Figure 6:**
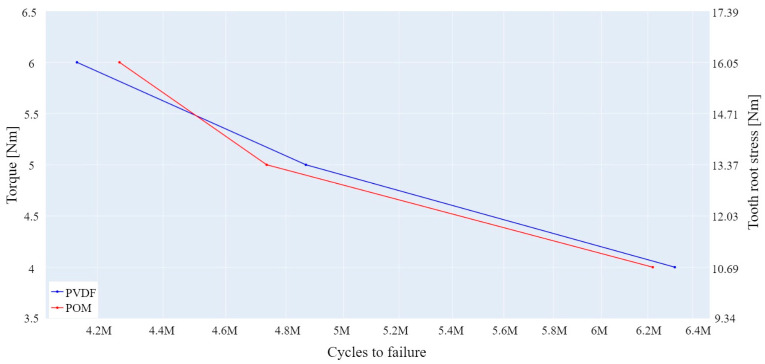
Lifespan results for the tested gear samples (90% survivability limit).

Figure 6 presents the lifespan curves for the tested PVDF and POM gear samples. The curves are established based on a 90% survival rate limit, which is often referred to as B10. This limit was determined by using the Weibull distribution, as it is a standard tool for failure prediction in long-term testing of a smaller number of samples [2,58]. The necessary shape and scale parameters, β and η, respectively, were determined with the MINITAB v20.4 software. Table 8 provides a summary of Weibull parameters along with a 90% survivability limit for tested gear samples.

Figure 6 and Table 8 show lifespan comparisons of the PVDF and POM gears in a mesh with steel gear. As shown, both materials have similar lifespans under certain load conditions. At higher load levels, due to increased heat development, the wear rate is higher, which ultimately results in a shorter lifespan compared to lower load levels.

#### 3.2.3. Design Parameters

Table 9 presents the evaluated design parameters regarding tooth load capacity and estimated coefficients of friction for the analyzed PVDF and POM gears mated with a steel pinion.

Due to different contact configurations, several different parameters were used to perform calculations according to the VDI 2736 guidelines. Different parameters used in Equations (1), (2), and (4) are:

- $Z_{EC45PVDF}=26.8$—Elasticity factor for steel/PVDF gear configuration;
- $Z_{EC45POM}=31.65$—Elasticity factor for steel/POM configuration;
- $k_{\vartheta ,Fu\beta }=900\frac{K\cdot {\left(\frac{m}{s}\right)}^{0.75}\cdot {mm}^{1.75}}{W}$—Heat transfer coefficient.

#### 3.2.4. Wear Analysis

The VDI 2736 guidelines propose a wear control method that describes tooth thickness reduction, i.e., linear wear, according to Equation (5). Averaged linear wear is the physical size used to determine, describe, and correlate the wear of materials in different applications and load conditions.

(5) $$W_{m}=\frac{T_{d}\cdot 2\cdot \pi \cdot N_{L}\cdot H_{V}\cdot k_{w}}{b_{w}\cdot z\cdot l_{Fl}}\leq W_{zul}.$$

The current version of the VDI 2736 is quite limited in terms of wear control since it contains wear coefficients $k_{w}$ only for POM and PBT materials [15]. The necessary wear coefficients can be obtained utilizing optical methods, i.e., a digital microscope, where the measured linear wear $W_{m}$ can be used to calculate the wear coefficient $k_{w}$. This method requires Equation (4) to be rewritten in the form presented in Equation (6):

(6) $$k_{w}=\frac{W_{m}\cdot b_{w}\cdot z\cdot l_{Fl}}{T_{d}\cdot 2\cdot \pi \cdot N_{L}\cdot H_{V}}.$$

Wear coefficients are calculated using the following parameters:

- $T_{d}$—Nominal torque (based on load level);
- $N_{L}$—Number of load cycles (based on lifespan);
- $l_{Fl}=6.67$ mm—Profile line length.

**Figure 7:**
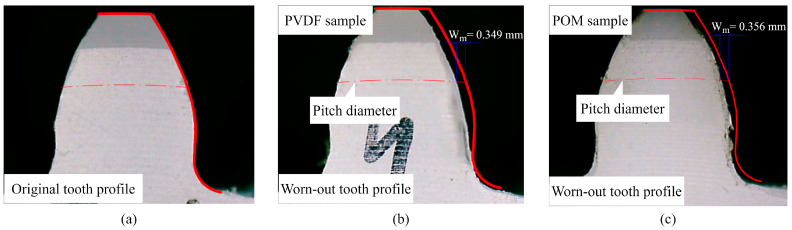
Tooth profile: (**a**) original tooth profile; (**b**) worn-out tooth profile for PVDF gear sample at 6 Nm load; (**c**) worn-out tooth profile for POM gear sample at 6 Nm load.

The omitted parameters were already used in previous gear calculations. The calculated wear coefficients for PVDF and POM samples are presented in Table 10. Figure 7a shows the original, i.e., unworn tooth profile. The procedure for evaluating the wear rate $W_{m}$ is shown in Figure 7b and Figure 7c for PVDF and POM gear samples, respectively.

The worn-out profiles of PVDF and POM gear samples are measured using the digital microscope (NB-MIKR-500) at a 22-fold magnification. The profiles are imported into the PortableCapture Plus 3.1 software, where the worn-out tooth profiles are compared to theoretical tooth geometry. Wear is determined by measuring the longest perpendicular distance between the theoretical and worn-out profiles [2,18]. The location of this distance is mainly focused around the pitch diameter, as shown in Figure 7b,c. Wear is measured for all of the teeth in the same gear sample, where the mean value of $W_{m}$ is taken as a reference value.

## 4. Discussion

This research investigates the potential of the PVDF polymer material in gear applications. Considering that the literature on this application of the PVDF material is rather sparse and there are currently no design parameters available for this application, comparative testing was performed on PVDF and POM gear samples. The conducted study demonstrates the potential of the PVDF material in gear applications, as the results of incremental load tests show significantly better properties of the PVDF material in self-mated engagement compared to POM, and lifespan tests show similar properties in the steel/PVDF engagement compared to steel/POM engagement.

The presented incremental load test procedure shows a unique way to quickly establish the necessary design parameters under various loading conditions. The key parameter necessary to establish reliable operational data presented in Table 7 is the temperature stability at each load level during the tests. The temperature stability criterion at a single load level used in this research was based on the following recommendations: The increase in monitored temperature at the tooth root after 90% of the single load step duration needs to be lower than 5 °C when compared to the average temperature monitored after 10% of the single load step duration [8]. All of the self-mated configurations in this research fulfilled the criterion, apart from the load levels, which included gear failure. It is important to notice here that the incremental load test temperature stability criteria are very generally given, and the previously explained criteria may vary due to polymer materials, gear sizes, load conditions, gear pair housing, etc. Also, a single load step duration of 2 × 10^5^ cycles used in this research is very generally introduced by other researchers [6,8]. Although the specified single load step duration was necessary to fulfill the temperature stability criterium and obtain the necessary design parameters, it is difficult to expect that this step duration is appropriate for higher rotational speeds, especially in cases of small plastic gears under low or moderate loads where, although the gears do not experience any failure mechanisms, the temperature stability is not achieved [6,8,26].

The coefficient of friction value of the self-mated POM configuration, presented in Table 7, which amounts to 0.16, shows a significant difference compared to the value for the same configuration specified in the VDI 2736 guideline, which amounts to 0.28 [15]. This shows the importance of gear pair testing at a certain load and specified geometry. The coefficient of friction specified in the guidelines amounts to 0.28 and was established for certain geometry and test conditions, i.e., through tribological testing, which does not correspond to the presented experimental setup. Using this value would lead to an uncertain gear design, which would significantly overestimate the necessary design parameters. Since no data can be found in the literature concerning the coefficient of friction of the PVDF gears, which amounts to 0.17, only a similarity of results can be established between self-mated PVDF and POM gears.

As shown in Table 9, the value of the coefficient of friction for the steel/PVDF engagement amounts to 0.17, which is lower than the value of 0.21 for the steel/POM engagement. Although the coefficient of friction value for the steel/PVDF is slightly lower, both of these values show good correspondence with the value specified very generally in the VDI 2736 guidelines for the dry steel/plastic pairing, which amounts to 0.2 [15]. Even though the value specified in the VDI 2736 guidelines is obtained using tribological tests, good correspondence of the results is prescribed to the metal countersurface, which neglects the thermal effects on the polymer material.

Although the coefficient of friction is tightly related to the gear bulk temperature, which has a significant effect on the wear rate of the polymer gear, the presented wear control method, suggested in the VDI 2736 guidelines, does not take into consideration the thermal effects on the mechanical properties of the polymer gears [19]. The wear coefficients presented in this research are in close correlation with the temperatures specified in Table 9. Wear coefficients for both steel/PVDF and steel/POM gears, shown in Table 10, show good correspondence at higher load levels. At the load level of 4 Nm, the steel/POM configuration shows better wear properties, which is explained by the time needed for the transfer layer to be formed at the steel/PVDF engagement. Once a transfer layer of molten material is formed, the wear rate decreases, as presented for higher loads of 5 Nm and 6 Nm. The wear coefficients presented in the VDI 2736 guideline are obtained from standard tribological tests, i.e., pin-on-disc, which can lead to uncertainty during the design process [59]. The guideline provides wear coefficients only for steel/POM and steel/PBT configurations, for two different surface roughnesses of the steel pinion flank. The proposed wear coefficient value of the steel/POM engagement with the surface roughness of 0.45 µm amounts to 1 × 10^−6^ mm^3^/(Nm), while the same engagement with the surface roughness of 1.5 µm has the proposed coefficient value of 3.4 × 10^−6^ mm^3^/(Nm) [15]. Both of these values differ noticeably from the values presented in Table 10 for the steel/POM engagement, which shows the significance of the wear coefficient determination for specific applications [60]. The wear coefficient values presented in Table 10 do not consider the roughness of the steel pinion flank, which represents a certain limitation regarding the conducted research.

The results presented in this research have a restriction on their use only for gear applications with operational conditions similar to the tested load values. Analyzing the presented results, it is obvious that the obtained design parameters vary for many reasons [61]. For example, the coefficient of friction and wear coefficient depend on the contact pressure distribution, relative sliding, sliding length, and temperature [62]. When analyzing the polymer gear with different geometry, it becomes evident these coefficients change. Considering that the polymer gear geometry in real-life applications is most likely different from the geometry of the tested gear samples, it is mandatory to experimentally test gears for each application. By utilizing experimental testing and evaluating the operational parameters with the described methods, it is possible to obtain reliable data for gear design [63].

There are a lot of directions for further research on this topic. Primarily, a broad investigation of numerous polymer materials in gear applications is necessary to provide relevant operational data for designers. Also, the testing methods for the self-mated contact of polymer gears should cover lifetime tests to provide a better understanding of these contact configurations at constant load conditions. The scope of this research did not cover this test configuration due to the tribological incompatibility of the self-mated POM, where any comparison with the PVDF gear samples would be inadequate. Future research could also be directed toward the development of a numerical simulation model of the wear processes that occur on the tooth flank [64]. Such a model would require a known wear coefficient for certain materials and would cover the basics of contact mechanics, i.e., the non-conformal contact of the tooth flank surfaces and changes in tribological limits that occur due to wear [65]. The model would be based on an iterative procedure that accounts for changes in flank surface topography, and based on that, calculates the changes in contact pressure, sliding conditions, and mesh stiffness to evaluate the sensitivity of various design parameters under certain load conditions.

## 5. Conclusions

The main conclusions of the presented research are the following:

1. The study reveals that self-mated PVDF gears fail due to thermal overload, particularly at higher load levels (9 Nm), where the frictional losses are severe. The study also reveals that the failure of the self-mated POM gears at 8 Nm is attributed to excessive wear, which is the reason this configuration is classified as tribologically incompatible. The study suggests that incremental load tests are crucial for establishing design parameters and endurance limits of polymer gears, reducing uncertainty, and avoiding critical operational conditions.
2. Experimental testing can provide reliable data for polymer gear design using temperature monitoring and optical methods to determine necessary design parameters. The VDI 2736 guidelines suggest using temperature monitoring at the tooth root area and a bulk temperature model to assess the coefficient of friction during tooth flank engagement. These values can be used to analyze different meshing configurations and load conditions, as well as to establish relevant tribological phenomena in polymer materials. The bulk temperature equation suggested by the VDI 2736 guidelines is used to determine the coefficients of friction. The self-mated contacts show similar coefficient values, but the self-mated POM contact develop even higher temperatures at the same load conditions. The steel/PVDF contact results in a lower coefficient of friction compared to the steel/POM engagement. The transfer film of molten PVDF material reduces frictional losses at the tooth contact. Higher coefficients of friction are expected at higher load levels due to the removal of the transfer layer. Although the values specified in the VDI 2736 guidelines show good agreement with the ones obtained by the experimental testing, the authors suggest that every new gear design should be determined using the presented methodology, primarily because of the broad range of gear sizes, materials, and load conditions, which may contribute to a significant deviation of results compared to the guidelines;
3. With the optical methods, i.e., the digital microscope, it is possible to determine the averaged linear wear and establish wear propagation at different numbers of working cycles. It is also possible to evaluate the necessary wear coefficients for different polymer materials at a certain load level and perform a comparative analysis of wear intensity. The wear coefficients are also determined based on the wear equation proposed in the VDI 2736 guidelines [15]. With the proper usage of optical methods, it is possible to reliably determine the necessary wear coefficients for specific applications and to have a reliable gear design as a result. The limited values specified in the VDI 2736 guidelines show a significant difference between the values obtained from the experimental research. This implies that standard tribological testing could lead to an unreliable design, as the wear characteristics may be overestimated.
4. Considering the lifetime test results obtained by the means of Weibull distribution deployed on five samples of each polymer/steel configuration, PVDF gears mated with steel pinion show similar lifespan properties compared to steel/POM mesh due to the formed transfer layer of molten PVDF material, which serves as a lubricant and has a positive effect on the wear properties. Due to increased wear rates at higher load levels, as expected, gear samples experience shorter lifetimes;
5. Considering numerous design parameters evaluated throughout the research and the lifespan curves obtained for the 90% survival rate (B10 limit), PVDF is an appropriate polymer material that can be used in gear applications. The latter conclusion applies both for self-mated contact and steel/PVDF contact, as the operational data obtained from experimental testing reveals superior properties of the self-mated PVDF gear samples compared to POM, and similar design parameters and lifespan results for steel/PVDF and steel/POM gears.

## Figures and Tables

**Table 1:** PVDF material properties.

Parameter	Value	Test Method
Density	1.78 g/cm^3^	ISO 1183 [45]
Tensile modulus ^1^	2000 MPa	ISO 527-2 [46]
Tensile strength ^1^	50 MPa	ISO 527-2
Coefficient of linear expansion ^2^	1.2⋅10^−4^ K^−1^	ISO 11359 [47]
Thermal conductivity ^2^	0.19 W/(K⋅m)	DIN 52612 [48]
Melting temperature ^3^	169 °C	ISO 3146 [49]

^1^ v = 50 mm/min; ^2^ at length, 23–100 °C; ^3^ DSC, 10 K/min.

**Table 2:** POM material properties.

Parameter	Value	Test Method
Density	1.41 g/cm^3^	ISO 1183
Tensile modulus ^1^	2800 MPa	ISO 527-2
Tensile strength ^1^	67 MPa	ISO 527-2
Coefficient of linear expansion ^2^	1.4⋅10^−4^ K^−1^	ISO 11359
Thermal conductivity ^2^	0.39 W/(K⋅m)	ISO 22007-4 [50]
Melting temperature ^3^	166 °C	DIN 53765 [51]

^1^ v = 1 mm/min; ^2^ at length, 23–100 °C; ^3^ DSC, 10 K/min.

**Table 3:** The main chemical properties of the PVDF and POM materials.

Parameter	PVDF	POM	Test Method
Water absorption	0.04%	0.04%	ISO 62-1 [52]
Oxygen index	43%	44%	ISO 4589-2 [53]
Flammability UL94	V0	V0	ISO 4589-2

**Table 4:** PVDF and POM material properties at 120 °C and heat deflection temperatures.

Parameter	PVDF		POM		Test Method
	Value (120 °C)	Value (150 °C)	Value (120 °C)	Value (140 °C)	
Density	1.08 g/cm^3^	0.7 g/cm^3^	0.97 g/cm^3^	0.59 g/cm^3^	ISO 1183
Tensile modulus	1400 MPa	714 MPa	1500 MPa	1050 MPa	ISO 527-2
Tensile strength	14.1 MPa	5.52 MPa	19.3 MPa	7.4 MPa	ISO 527-2

**Table 5:** Basic geometric parameters of the tested polymer gear samples.

Parameter	Value
Profile	Involute, ISO 53 A [56]
Module	3 mm
Number of teeth	17
Pressure angle	20°
The face width	20 mm
Coefficient of profile shift	0

**Table 6:** Basic testing parameters at the pitch diameter.

Parameter	Value
Revolutions per minute	1000 rpm
Linear velocity	2.67 m/s
Minimum specific sliding (17 teeth)	−6.66
Maximum specific sliding (17 teeth)	0.87

**Table 7:** Summary of incremental load tests at specific load levels.

**Material combination**	**Torque at failure (Nm)**	**Load level (Nm)**								**Average COF** **value**
		1	2	3	4	5	6	7	8	
		$\mathrm{Tooth}\;\mathrm{root}\;\mathrm{stress},\;\sigma_{F\;}$ **(MPa)**								
		2.67	5.35	8.02	10.69	13.37	16.05	18.72	21.39	
PVDF/PVDF	9	$\mathrm{Tooth}\;\mathrm{flank}\;\mathrm{pressure},\;\sigma_{H\;}$ **(MPa)**								0.17
		10.72	15.17	18.58	21.46	23.99	26.28	28.38	30.34	
		$\mathrm{Gear}\;\mathrm{bulk}\;\mathrm{temperature},\;\vartheta_{Fu\beta \;}$ **(°C)**								
		40.08	45.84	52.68	60.12	68.76	80.64	93.01	111.6	
POM/POM	8	$\mathrm{Tooth}\;\mathrm{flank}\;\mathrm{pressure},\;\sigma_{H\;}$ **(MPa)**								0.16
		12.69	17.95	21.99	25.39	28.38	31.09	33.58	/	
		$\mathrm{Gear}\;\mathrm{bulk}\;\mathrm{temperature},\;\vartheta_{Fu\beta \;}$ **(°C)**								
		40.44	42.84	56.57	64.44	76.56	88.16	119.04	/	

**Table 8:** Weibull distribution parameters.

**Material combination**	**Load level (Nm)**		
	4	5	6
C45/PVDF	**The shape parameter,** β		
	5.97905	7.152	8.86897
	**The scale parameter,** η		
	5,496,841	4,332,340	3,768,810
	90% survival rate limit		
	6,319,664	4,868,194	4,140,425
C45/POM	**The shape parameter,** β		
	1.18451	11.0874	7.90954
	**The scale parameter,** η		
	4,163,377	4,391,966	3,839,839
	**90% survival rate limit**		
	6,221,947	4,735,088	4,266,855

**Table 9:** Summary of lifetime tests.

**Material combination**	**Load level (Nm)**			**Average COF value**
	4	5	6	
	**Tooth root stress,** $\sigma_{F\;}$**(MPa)**			
	10.69	13.37	16.05	
C45/PVDF	**Tooth flank pressure,** $\sigma_{H\;}$**(MPa)**			0.17
	30.19	33.75	36.97	
	**Gear bulk temperature,** $\vartheta_{Fu\beta \;}$**(°C)**			
	43.4	52.7	69.6	
C45/POM	**Tooth flank pressure,** $\sigma_{H\;}$**(MPa)**			0.21
	35.65	39.86	43.66	
	**Gear bulk temperature,** $\vartheta_{Fu\beta }\;$**(°C)**			
	52.8	57.1	64.7	

**Table 10:** Evaluated wear coefficients.

**Material combination**	**Load level (Nm)**		
	4	5	6
C45/PVDF	$\mathrm{The}\;\mathrm{wear}\;\mathrm{coefficient},\;k_{w\;}$ **(10^−6^ mm^3^/(Nm))**		
	7.72	10.98	12.62
C45/POM	$\mathrm{The}\;\mathrm{wear}\;\mathrm{coefficient},\;k_{w\;}$ **(10^−6^ mm^3^/(Nm))**		
	5.58	9.77	12.96

## Data Availability

The data presented in this study are available on request from the main author.
